# The *bamA* gene for anaerobic ring fission is widely distributed in the environment

**DOI:** 10.3389/fmicb.2013.00302

**Published:** 2013-10-10

**Authors:** Abigail W. Porter, Lily Y. Young

**Affiliations:** Department of Environmental Science, School of Biological and Environmental Sciences, Rutgers UniversityNew Brunswick, NJ, USA

**Keywords:** anaerobic benzoyl-CoA pathway, *bamA* gene, 6-oxocylcohex-1-ene-1-carbonyl-CoA hydrolase, monoaromatic degradation, anaerobic hydrocarbon biodegradation

## Abstract

Benzoyl-CoA is the signature central metabolite associated with the anaerobic metabolism of a diverse range of compounds such as humic acid, lignin, amino acids, and industrial chemicals. Aromatic chemicals with different upstream degradation pathways all funnel into the downstream benzoyl-CoA pathway. Different genes encoding enzymes of the benzoyl-CoA pathway could be used as biomarkers for the anaerobic benzoyl-CoA pathway, however, the ring opening hydrolase, encoded by the *bamA* gene, is ideal because it is detected under a range of respiratory conditions, including under denitrifying, iron-reducing, sulfate-reducing, and fermentative conditions. This work evaluated DNA samples from six diverse environments for the presence of the *bamA* gene, and had positive results for every sample. Individual *bamA* gene clones from these sites were compared to published genome sequences. The clone sequences were distributed amongst the genome sequences, although there were clone sequences from two of the analyzed sites that formed a unique clade. Clone sequences were then grouped by site and analyzed with a functional operational taxonomic unit based clustering program to compare the *bamA* gene diversity of these sites to that of several locations reported in the literature. The results showed that the sequence diversity of the sites separated into two clusters, but there was no clear trend that could be related to the site characteristics. Interestingly, two pristine freshwater sites formed a subgroup within one of the larger clusters. Thus far the *bamA* gene has only been examined within the context of contaminated environments, however, this study demonstrates that the *bamA* gene is also detected in uncontaminated sites. The widespread presence of the *bamA* gene in diverse environments suggests that the anaerobic benzoyl-CoA pathway plays an important role in the global carbon cycle that has thus far been understudied.

## INTRODUCTION

**FIGURE 1:**
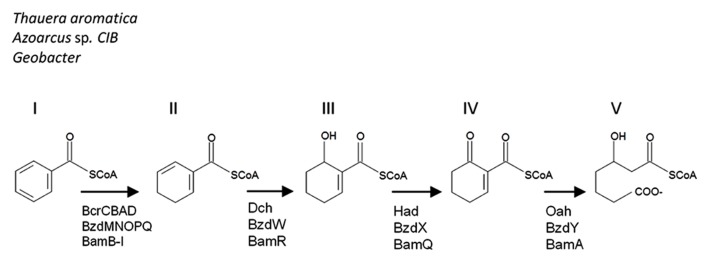
** A comparison of the benzoyl-CoA downstream metabolic pathways.** Benzoyl-CoA (I) is dearomatized to cyclohexa- 1,5-diene-1-carbonyl-CoA (II). For *Thauera, Azoarcus*, and *Geobacter* the metabolites include 6-hydroxycyclohex-1-ene-1-carbonyl-CoA (III), 6-oxocyclohex-1-ene-1-carbonyl-CoA (IV), and result in 3-hydroxypimelyl- CoA (V). The corresponding enzymes are indicated below the arrows in the pathway. The pathway is modified from Carmona et al. (2009).

Aromatic compounds are commonly found in the environment as components in petroleum, industrial contaminants, amino acids, or as humic acids and lignins. Due to the stability of the aromatic ring, these chemicals can be recalcitrant in the environment. Under aerobic conditions, molecular oxygen is incorporated into the aromatic structure by oxygenase enzymes. In the absence of oxygen, therefore, anaerobes have had to evolve different strategies in order to attack these aromatic rings and utilize the available carbon. While anaerobic monoaromatic compound degradation can be initiated in a number of different ways, the signature central metabolite for these pathways is benzoyl-CoA. Under anaerobic conditions, benzoyl-CoA degradation involves an initial systematic reduction of the benzene ring (**Figure 1**, structures II and III), followed by the hydrolysis of 6-oxocyclohex-1-ene-1-carbonyl-CoA (**Figure 1**, structure IV) to 3-hydroxypimelyl-CoA (**Figure 1**, structure V) by 6-oxocyclohex-1-ene-1-carbonyl-CoA hydrolase. This latter enzyme is encoded by the *bamA* gene and its homologs *oah* and *bzdY*, which were previously described in *Thauera aromatica* (Laempe et al., 1999) and *Azoarcus* strain CIB (López Barragán et al., 2004), respectively. These enzymes have the same function, but differ in their genetic nomenclature.

In contrast to the benzoyl-CoA reductase, which differs in obligate and facultative anaerobes (Boll and Fuchs, 1995; Peters et al., 2004; Hosoda et al., 2005; Song and Ward, 2005; Wischgoll et al., 2005), the *bamA* gene has been reported to be present in isolates growing under a range of redox conditions (Butler et al., 2007; McInerney et al., 2007; Kuntze et al., 2008; for review, see Carmona et al., 2009). Since regions of the *bamA* gene are highly conserved in a variety of different organisms that are known to utilize the benzoyl-CoA pathway, it is an ideal candidate for use as a molecular biomarker for anaerobic aromatic hydrocarbon biodegradation (Kuntze et al., 2008). In addition, focusing on the *bamA* gene has the further benefit of indicating that the aromatic ring has been anaerobically cleaved, whereas the upstream reactions do not confer ring fission information.

Reports in the literature have suggested the potential usefulness of using a genetic biomarker based on the benzoyl-CoA pathway for benzene, toluene, and xylene (BTX) or other aromatic contaminants (Hosoda et al., 2005; Song and Ward, 2005; Kuntze et al., 2011; Staats et al., 2011; Li et al., 2012; Sun et al., 2013) yet this pathway is not exclusive to contaminants. There are other aromatic compounds that are found in or released into the environment, including vanillin, *p*-cresol, phenol, aniline, and phenylalanine that are also metabolized via benzoyl-CoA (Harwood et al., 1998).

The *bamA* gene has thus far been examined only in the context of BTX contamination. It has been used as a molecular biomarker in the laboratory to examine the functional microbial diversity in *p*-xylene degrading marine enrichments (Higashioka et al., 2011), in benzoate- and toluene-degrading denitrifying freshwater enrichments (Li et al., 2012), and in sulfate-reducing, denitrifying, and methanogenic toluene-degrading microcosms (Sun et al., 2013). While the *bamA* gene has been identified in some aromatic compound-degrading isolates, there are a very limited number of studies in which the *bamA* gene has been used as an environmental biomarker. These include environmental surveys of benzene-contaminated aquifer samples (Kuntze et al., 2011), benzene-contaminated landfill leachate (Staats et al., 2011), and crude oil-contaminated mangrove sediment (Andrade et al., 2012).

The *bamA* gene has not yet been widely detected in the environment because it has only been evaluated under very limited conditions. We hypothesize that since the *bamA* gene is specific for a common anaerobic pathway that is would also be key to the downstream metabolism of other types of aromatic compounds, including naturally occurring substrates, and thus be present in uncontaminated sites. This study used culture-independent techniques to examine the *bamA* gene diversity in DNA extracts from a range of environments and habitats, both with and without aromatic hydrocarbon contaminants, to examine the range of environments in which this gene could be detected as a proxy for the presence of the anaerobic benzoyl-CoA pathway.

## MATERIALS AND METHODS

### DNA EXTRACTIONS

DNA was extracted from a variety of environments and sample types as described in **Table 1**. Specifically, DNA was extracted from 0.5 g sediment samples from sites in New Jersey, USA, and from 1 g of estuarine and marine sediments from Massachusetts, USA, using a PowerSoil DNA Extraction Kit (Mo Bio Laboratories, Carlsbad, CA) as per the manufacturer’s instructions. Approximately 300 ml groundwater samples from Six Mile Run and Crosswicks Creek were filtered through a 0.45 μm filter, which was cut into six pieces and extracted using an UltraClean Soil DNA Extraction Kit (Mo Bio Laboratories) with modifications to the manufacturer’s instructions as previously described (Barringer et al., 2010).

**Table 1:** Sites that have tested positive for the *bamA* gene grouped by the states in which the sites are located.

Location	Sample type	Site description	Available electron acceptors	Reference
***New Jersey***
Crosswicks Creek	Groundwater	Agricultural impact	Fe^3+^	Barringer et al. (2010)
Joint Meetings of Essex and Union Counties Wastewater Treatment Facility	Digester sludge	Anaerobic digester	CO_2_	Crawford et al. (1995)
Mashipacong Bogs Preserve	Sediment	Pristine peat bog	CO_2_	This study
Six Mile Run	Groundwater	No contamination	Fe^3+^	This study
***Massachusetts***
Eel Pond	Sediment	Estuarine harbor with frequent boat activity	${SO}_{4}^{2-}$	This study
Wild Harbor	Sediment	Historic petroleum contamination	${SO}_{4}^{2-}$	Teal et al. (1978); Culbertson et al. (2007)

*The type of sample used in the DNA extraction is indicated, as is the site description which includes potential chemical inputs.*

A conserved 300 base pair region of the *bamA* gene was targeted with PCR primers BamA SP9F (5′ CAG TAC AAY TCC TAC ACV ACB G 3′) and BamA ASP1R (5′ CMA TGC CGA TYT CCT GRC 3′) as previously described by Kuntze et al. (2008). Each 20 μl reaction included 10 mg bovine serum albumin (New England Biolabs, Ipswich, MA, USA), 40 pmol forward and reverse primers, 0.5 mM MgCl_2_, 0.2 mM dNTPs, and 1 U REDTaq DNA polymerase. The reaction conditions were 5 min at 94°C, followed by 35 cycles of denaturation at 94°C for 30 s, annealing at 59°C for 45 s, and elongation at 72°C for 60 s, and concluded at 72°C for 10 min. All reaction components were from Sigma-Aldrich (St. Louis, MO, USA) unless otherwise indicated.

An arsenic-reducing isolate, Strain MPA-C3, was used as a negative control in the PCR assay. This strain was isolated in our laboratory and had demonstrated anaerobic benzoate-degrading activity, although genome analysis has shown that it does not contain a *bamA* homolog (Mumford et al., 2013). Strain MPA-C3 was grown in anaerobic freshwater minimal medium (Mumford et al., 2012) with 2 mM NaH_2_AsO_4_ as the terminal electron acceptor and 2 mM sodium acetate as the electron donor. DNA was extracted from 1 ml of log phase culture using the Mo Bio UltraClean DNA extraction kit, according to the manufacturer’s instruction (Mumford et al., 2013).

The *bamA* gene diversity was further explored in DNA samples from Six Mile Run, Crosswicks Creek, anaerobic digester sludge, and a peat bog by cloning the gene into pGEM-T Easy (Promega, Madison, WI, USA). The Eel Pond and Wild Harbor *bamA* gene PCR products were cloned into an Invitrogen TOPO TA cloning vector (Life Technologies, Grand Island, NY, USA). We randomly selected 38 Eel Pond, 42 Wild Harbor, 8 Crosswicks Creek, 6 Six Mile Run, 6 anaerobic digester, and 13 peat bog clones for nucleotide sequencing (Genewiz, Plainfield, NJ, USA).

### PREDICTED AMINO ACID SEQUENCE ANALYSIS

Predicted amino acid sequences were aligned with ClustalX (Larkin et al., 2007) with BamA hydrolase amino acid sequences from genome sequences and biochemically characterized isolates that have demonstrated anaerobic aromatic hydrocarbon degradation activity. Using ClustalX, we generated a bootstrapped neighbor-joined tree, using 1000 iterations, with methyl-6-ketocyclohex-1-ene-1-carbonyl-CoA hydrolase (accession number CCH23022.1) as the outgroup.

Functional operational taxonomic unit (OTU) analyses with the Mothur software package (Schloss et al., 2009) were used to compare site-dependent diversity. The analysis included the clones described above with sequences that had been deposited in GenBank from environmental analyses of 192 clones from Banisveld Landfill (Staats et al., 2011), 6 clones from Hansemann Aquifer (Kuntze et al., 2011), and 9 clones from Gneisenau Aquifer (Kuntze et al., 2011).

### NUCLEOTIDE SEQUENCES

Nucleotide sequences were deposited in GenBank under accession numbers KF170933–KF171005.

## RESULTS

All the DNA extracts from the diverse environmental habitats listed in **Table 1** tested positive for the *bamA* gene. These sites included Crosswicks Creek and Six Mile Run, both of which are uncontaminated, iron rich environments, as well as methanogenic sites including sediment from a pristine bog and sludge from an anaerobic digester. Additionally, two petroleum-contaminated sites Wild Harbor and Eel Pond, which are marine and estuarine, respectively, also contained the *bamA* gene. We cloned and sequenced random clones from these environments.

**FIGURE 2:**
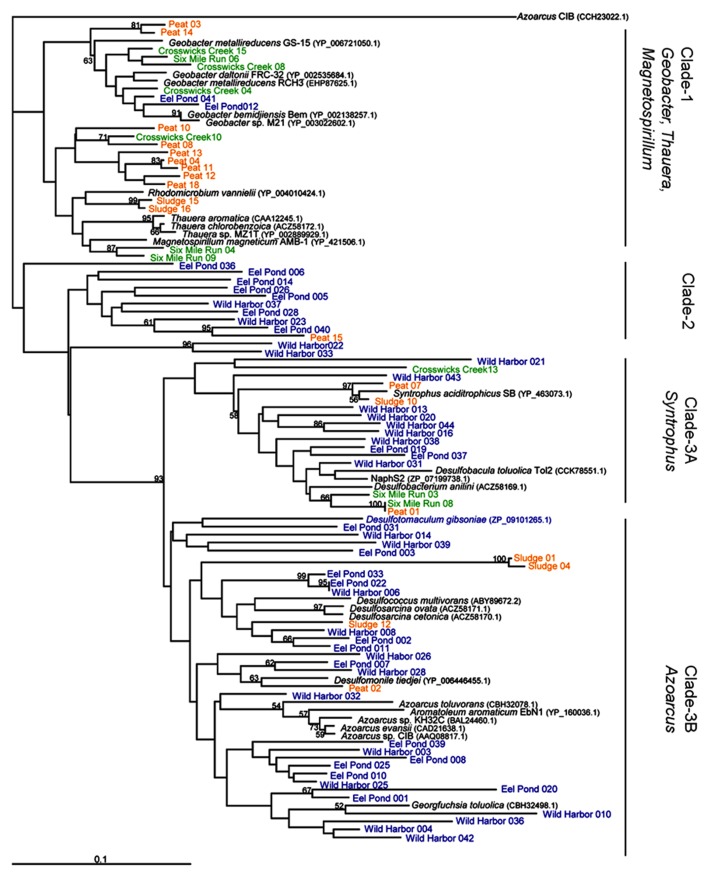
**BamA amino acid sequence from characterized isolates, genome sequences, and environmental clones.** This is a neighbor-joining tree based on a 97 amino acid sequence alignment and rooted to *Azoarcus* sp. CIB methyl-6-ketocyclohex-1-ene-1-carbonyl-CoA hydrolase. Bootstrap values are given for 1000 replicate trees, and the values greater than 50% are indicated. Clones in blue indicate marine sediment DNA extracts (Eel Pond and Wild Harbor), green indicates groundwater samples (Crosswicks Creek and Six Mile Run), and orange represents methanogenic sites (anaerobic digester and peat bog). Clade-1 contains genome sequences from *Geobacter*, *Magnetospirillum*, and *Thauera* sp. Clade-2 is unique to this study and only contains sequences from Eel Pond and Wild Harbor. Clade-3 has representatives from the *Syntrophus* (Clade-3A) and *Azoarcus* (Clade-3B) isolates, along with a several sulfate-reducing microorganisms.

To compare these new *bamA* gene sequences with those previously reported in the literature, we constructed a neighbor-joined tree of predicted amino acid sequences from characterized anaerobic aromatic hydrocarbon-degrading isolates and genome sequences, in addition to our environmental clones, as shown in **Figure 2**. There were three major clades. Clade-1 grouped the BamA amino acid sequences from *Rhodomicrobium vannielii* with the previously established grouping of *Geobacter/Magnetospirillum/Thauera* (Staats et al., 2011). More specifically, this clade also included the Oah amino acid sequence from *T. aromatica *(accession number CAA12245), which clustered closely with sequences from two other *Thauera* isolates. The *T. aromatica* Oah amino acid sequence shared 82% identity with the *Geobacter bemidjiensis* Bem BamA amino acid sequence (accession number YP_002138257) over the 100 residue region analyzed in this study. As with the sequences from *Thauera* isolates, the BamA amino acid sequences from *Geobacter* genomes clustered together to form a separate grouping within Clade-1, although this grouping also included sequences from our Crosswicks Creek and Six Mile Run groundwater samples (**Figure 2**, Clade-1).

Interestingly, we found that Clade-2 (**Figure 2**) was almost exclusively composed of clones from marine environments Eel Pond and Wild Harbor, with a single clone sequence from the peat bog DNA extract. The sequences in Clade-2 did not cluster with any known characterized isolates or genome sequences, although this may be due to the limited number of BamA hydrolase sequences available. Wild Harbor clone sequences were found exclusively in Clades 2 and 3, whereas the clones from Eel Pond were distributed throughout the dendrogram.

The third clade contained the *Syntrophus* and *Azoarcus* groups (Clade-3A and Clade-3B, respectively). The *Azoarcus* group included the *Azoarcus* strain CIB BzdY (accession number AAQ08817) amino acid sequence, which also clustered closely with the BamA amino acid sequence from other *Azoarcus* isolates (**Figure 2**). The *G. bemidjiensis* Bem BamA sequence from above and the *Azoarcus* strain CIB BzdY amino acid sequence shared 70% identity for the 100 residue region analyzed in this study, which further illustrates the differences between clades. Clade-3 also included a number sequences from sulfate-reducing organisms, such as *Desulfobacterium anilini*, *Desulfobacula toluolica* tol2, and NaphS2, that were distributed in both Clade-3A and Clade-3B. Wild Harbor and Eel Pond clones were present in both Clade-3A and Clade-3B as well, as were clones from the anaerobic digester and peat bog DNA samples. The BamA hydrolase sequences from Crosswicks Creek and Six Mile Run groundwater samples were found only in Clade-3A.

**FIGURE 3:**
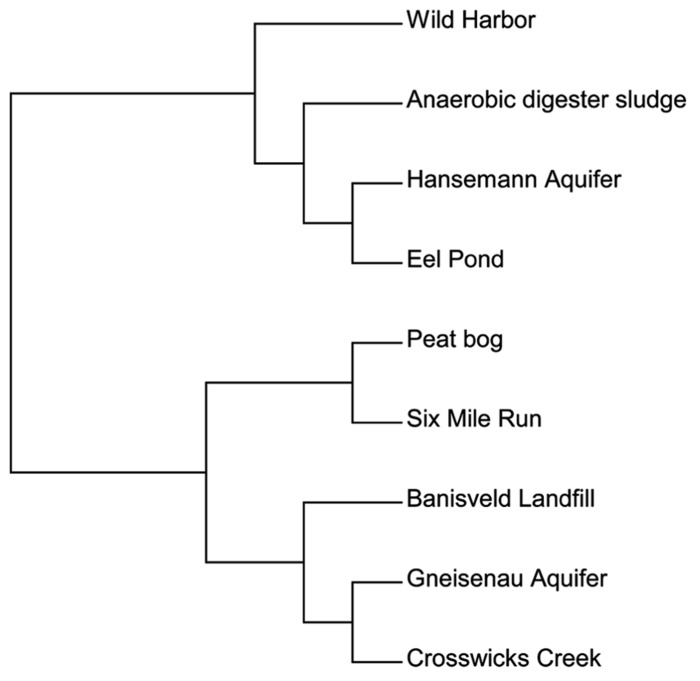
**A functional OTU-based cluster analysis of environmental samples based on location.** Amino acid sequences from all published culture-independent surveys were compared over an 81 amino acid region of the BamA hydrolase. Sites were compared using mothur software (Schloss et al., 2009) with a 95% cutoff.

We then used a different approach to examine the total *bamA* gene diversity within each site. In addition to our clone sequences, we also included sequence data from published environmental studies of Hansemann Aquifer and Gneisenau Aquifer, two benzene-contaminated freshwater locations, and Banisveld Landfill leachate, which was also a freshwater site contaminated with BTX chemicals (Kuntze et al., 2011; Staats et al., 2011). The diversity within each site was compared utilizing a functional OTU-based cluster analysis of the predicted amino acid sequences described above using Mothur software (Schloss et al., 2009). All of the sequences used here were from environmental DNA extracts and were not from laboratory cultures. **Figure 3** illustrates the differences in diversity across the different sites and shows that the sites were divided into two groups. This data set cannot be compared with the data set used in **Figure 2** in which individual sequences are compared to each other, as **Figure 3** shows the relationship between sites based on the diversity of the total sequences from each site.

The first group in **Figure 3** contained one cluster of BamA hydrolase sequences from sites that included Wild Harbor, anaerobic digester sludge, Hansemann Aquifer, and Eel Pond. Both Wild Harbor and Eel Pond were sites that have had long-term petroleum contamination, while the Hansemann Aquifer had benzene contamination (Kuntze et al., 2011). The anaerobic digester sludge likely contained other types of aromatic compounds and was not limited to petroleum hydrocarbon contamination. The second group was further divided into two different clusters. The first cluster grouped the total sequences from the peat bog and Six Mile Run groundwater clones together, both of which are clean sites and neither of which have petroleum contamination. Interestingly, these two sites are distinct from the second clustering of sequences from Banisveld Landfill leachate, the Gneisenau Aquifer, and Crosswicks Creek, although Gneisenau Aquifer and Crosswicks Creek shared more total sequence similarity to each other than to Banisveld Landfill sequences. While the Banisveld Landfill and Gneisenau Aquifer were reported to have benzene contamination (Kuntze et al., 2011; Staats et al., 2011), Crosswicks Creek was influenced by agricultural activity (Barringer et al., 2010). Based on our analysis of a limited number of clones from environmental samples, there is evidence of *bamA* gene diversity in the environment. Although there does not appear to be a clear pattern with respect to the total sequence diversity at the site level that can be attributed to the type of possible organic substrates at the site, it is interesting that the sequences from the peat bog and Six Mile Run, both uncontaminated freshwater sites, clustered separately.

## DISCUSSION

Our analysis of the individual BamA hydrolase amino acid sequence diversity is based upon environmental clones from our laboratory and from the limited number of characterized anaerobic aromatic hydrocarbon-degrading isolates. We did not include other environmental clones or enrichment culture sequences from other studies in **Figure 2**, as there was no clear association between the BamA amino acid sequence and metabolic activity from these complex samples. The results suggest that to some degree there is an association between some individual BamA hydrolase amino acid sequences and the sample site characteristics. For example, Crosswicks Creek clones are found mainly in Clade-1, which is in agreement with published work that detected measurable iron at the site and identified 16S rRNA gene clones with high similarity to *Geobacter* sp. Ply1 (Mumford et al., 2012). The peat bog and anaerobic digester clone sequences originated from methanogenic environments and are predominately found in Clade-1 (**Figure 2**). There are sequences, nonetheless, from these clones that are also found in Clade-3 and cluster near *Syntrophus aciditrophicus*, a fermenting benzoate-degrading organism (McInerney et al., 2007). *Syntrophus*-like organisms have been previously identified in anaerobic digester sludge (Shin et al., 2010).

The majority of the Eel Pond and Wild Harbor sequences are in Clade-2 and Clade-3 (see **Figure 2**). In addition, Clade-3A and Clade-3B both contain BamA hydrolase sequences from genome sequences of sulfate-reducing microorganisms. The clustering of sequences from Eel Pond and Wild Harbor is not unexpected given that they are both coastal marine sites with abundant sulfate that would be found in seawater, and thus support sulfate reducers. The wider distribution of the Eel Pond sequences (Clade-1,-2, and -3), however, differ from the somewhat more narrow distribution of Wild Harbor sequences. Sun et al. (2013) suggested a correlation between the *bamA* gene sequence and the terminal electron acceptor that was supplied in their microcosm study. That does appear to be the case for the BamA hydrolase sequences from isolates, since the sequences from a particular genus clustered together as previously reported (Kuntze et al., 2008, 2011; Staats et al., 2011). Given the small number of BamA hydrolase sequences available from isolated organisms with available genomes, however, it is difficult to say with confidence that the clone sequences are derived from a specific group within our samples.

In addition to examining the individual clone sequences, we completed a different analysis in which the sequences were grouped by site and the differences in total BamA hydrolase amino acid sequence diversity were examined to see if there was a trend between the study sites and the BamA hydrolase diversity. We included published environmental sequences, although we chose to focus only on the sites with publicly available sequences that did not originate from laboratory cultures (Kuntze et al., 2011; Staats et al., 2011). While there have been studies that examined *bamA* gene diversity in enrichments or microcosms, the culturing process would have favored specific organisms that were able to utilize the exogenous substrate and thus would not provide an accurate picture of the *bamA* gene sequences that were present at the site from which the inocula came (Higashioka et al., 2011; Li et al., 2012; Sun et al., 2013). Our intent was to study BamA hydrolase diversity in the natural habitat without culturing or enrichment. The results of site-based diversity comparisons suggest that there were not differences in diversity with respect to type of sites and potentially available organic substrates. For example, both Eel Pond and Wild Harbor are marine sites with historical petroleum contamination. While the total sequences from these two associated together in the same large clustering, the Eel Pond sequence diversity was more similar to that of the benzene-contaminated freshwater Gneisenau Aquifer than to Wild Harbor sequences. Similarly, benzene-contaminated freshwater sites (Banisveld Landfill, Gneisenau Aquifer, and Hansemann Aquifer) were present in both clusters. In contrast, it is interesting that the total sequences from the peat bog and Six Mile Run, both uncontaminated freshwater sites, appear to cluster separately from the other sites. It should be noted, however, that the data presented here are from a limited number of clones in addition to only three sites in the literature from which data were available. The number of available sequences was less than 50 for all of our sites, the Gneisenau Aquifer, and the Hansemann Aquifer (Kuntze et al., 2011), although there were nearly 200 sequences from the Banisveld Landfill (Staats et al., 2011). Expanding the number of sample sites and increasing the sequence coverage with next generation sequencing techniques could potentially yield a more complete assessment of *bamA* gene diversity in the environment by capturing the less abundant sequences that might be overlooked in cloning experiments.

A large number of naturally occurring aromatic compounds, including amino acids, humic acid, plant phenolics, and lignin metabolites, are metabolized through the anaerobic benzoyl-CoA pathway, therefore the benzoyl-CoA pathway must play an important role in global anaerobic aromatic hydrocarbon metabolism. We detected the *bamA* gene in petroleum-contaminated marine sediments, uncontaminated groundwater, anaerobic digester sludge, and peat bog sediment, suggesting that the benzoyl-CoA pathway is common in the environment. Our findings are in agreement with a previous study (Sun et al., 2013) that detected the *bamA* gene in toluene amended microcosms from diverse inocula, including contaminated sediment and soil, anaerobic digester sludge, aerobic digester sludge, and uncontaminated soil. More specifically, the data reported here demonstrates that the BamA hydrolase is a common feature for the downstream metabolism of the key benzoyl-CoA pathway in anaerobic environments. This, however, does not necessarily relate to contaminant presence, but rather could be the contribution of natural aromatic inputs such as lignin and humic acid. For example, lignin metabolites identified in methanogenic enrichment cultures include cinnamic, vanillic, syringic, and ferulic acids (Colberg and Young, 1985), which would also be metabolized through the benzoyl-CoA pathway. Since the *bamA* gene has not yet been studied in the context of natural aromatic substrates, future work should utilize a quantitative approach, such as qPCR, to elucidate the naturally occurring abundance of the *bamA* gene in the environment.

The *bamA* gene is a biomarker for the anaerobic benzoyl-CoA pathway, and more broadly anaerobic aromatic hydrocarbon degradation, that we detected in both contaminated and pristine environments. In addition, the *bamA* gene could be used as a tool for examining sites that contain chemicals with either unknown or multiple mechanisms by which the substrate is anaerobically transformed to benzoyl-CoA. There is great diversity in the mechanisms used in the initial steps of anaerobic aromatic compound degradation that occur prior to benzoyl-CoA formation. For example, L-phenylalanine degradation requires an amino transferase to form phenylpyruvate, which further undergoes a series of steps including decarboxylation, and results in the ultimate formation of benzoyl-CoA (Schneider et al., 1997). In contrast, it has been widely reported that toluene degradation occurs through a fumarate addition mechanism, which results in benzylsuccinate as an intermediate prior to benzoyl-CoA formation (Evans et al., 1992; Biegert et al., 1996). On the other hand, the activation mechanism for anaerobic benzene degradation is less clear as three different mechanisms have been reported. One is methylation to form toluene, which is then metabolized via fumarate addition to benzylsuccinate (Ulrich et al., 2005). Secondly, benzene can undergo hydroxylation to form phenol, which can be further transformed to benzoate and subsequently benzoyl-CoA (Caldwell and Suflita, 2000; Chakraborty and Coates, 2005). Finally, direct carboxylation of benzene to benzoate has also been reported (Phelps et al., 2001; Kunapuli et al., 2008; Abu Laban et al., 2010). As a result, the *bamA* gene has been targeted, and consistently detected, in benzene-contaminated sites (Kuntze et al., 2011; Staats et al., 2011; Andrade et al., 2012). The *bamA* gene has demonstrated usefulness when used in conjunction with biomarkers for other genes in the benzoyl-CoA pathway (Kuntze et al., 2011) or with molecular probes for specific degradation steps that occur prior to benzoyl-CoA formation, such as the *bssA* gene which is key in toluene degradation (Staats et al., 2011; Andrade et al., 2012; Sun et al., 2013).

The anaerobic degradation of aromatic hydrocarbons, however, is more important than just with respect to contaminants. The cycling of carbon in anoxic environments is critical to the global carbon cycle but it receives less attention than in its oxic counterpart. To better utilize the *bamA* gene as a biomarker for anaerobic aromatic compound degradation through the benzoyl-CoA pathway, however, a more comprehensive set of data are needed for anaerobic degradation of naturally occurring aromatic compounds such as plant phenolics, humics, and lignin. Whether there are *bamA* gene differences for anthropogenic or natural aromatic substrates has yet to be determined. Nonetheless, evidence thus far, indicates that the *bamA* gene in the benzoyl-CoA pathway is central to anaerobic aromatic ring fission in a variety of environments and by extension is a key component in the global carbon cycle.

## Conflict of Interest Statement

The authors declare that the research was conducted in the absence of any commercial or financial relationships that could be construed as a potential conflict of interest.
